# Novel manifestations of Warburg micro syndrome type 1 caused by a new splicing variant of *RAB3GAP1*: a case report

**DOI:** 10.1186/s12883-021-02204-w

**Published:** 2021-04-28

**Authors:** Raziyeh Khalesi, Ehsan Razmara, Golareh Asgaritarghi, Ali Reza Tavasoli, Yasser Riazalhosseini, Daniel Auld, Masoud Garshasbi

**Affiliations:** 1 Department of Medical Genetics, Faculty of Medical Sciences, Tarbiat Modares University, Tehran, Iran; 2 Department of Genetics, Faculty of Biological Sciences, Tarbiat Modares University, Tehran, Iran; 3 Myelin Disorders Clinic, Pediatric Neurology Division, Children’s Medical Center, Pediatrics Center of Excellence, Tehran University of Medical Sciences, Tehran, Iran; 4 McGill Genome Centre, Montréal, Québec Canada; 5 Department of Human Genetics, McGill University, Montréal, Québec Canada

**Keywords:** PCR-RFLP, RAB3GAP1, Tetra-primer ARMS-PCR, WARBM type 1, Whole-exome sequencing

## Abstract

**Background:**

The present study aimed to determine the underlying genetic factors causing the possible Warburg micro syndrome (WARBM) phenotype in two Iranian patients.

**Case presentation:**

A 5-year-old female and a 4.5-year-old male were referred due to microcephaly, global developmental delay, and dysmorphic features. After doing neuroimaging and clinical examinations, due to the heterogeneity of neurodevelopmental disorders, we subjected 7 family members to whole-exome sequencing. Three candidate variants were confirmed by Sanger sequencing and allele frequency of each variant was also determined in 300 healthy ethnically matched people using the tetra-primer amplification refractory mutation system-PCR and PCR-restriction fragment length polymorphism. To show the splicing effects, reverse transcription-PCR (RT-PCR) and RT-qPCR were performed, followed by Sanger sequencing. A novel homozygous variant—NM_012233.2: c.151-5 T > G; p.(Gly51IlefsTer15)—in the *RAB3GAP1* gene was identified as the most likely disease-causing variant. RT-PCR/RT-qPCR showed that this variant can activate a cryptic site of splicing in intron 3, changing the splicing and gene expression processes. We also identified some novel manifestations in association with WARBM type 1 to touch upon abnormal philtrum, prominent antitragus, downturned corners of the mouth, malaligned teeth, scrotal hypoplasia, low anterior hairline, hypertrichosis of upper back, spastic diplegia to quadriplegia, and cerebral white matter signal changes.

**Conclusions:**

Due to the common phenotypes between WARBMs and Martsolf syndrome (MIM: 212720), we suggest using the “RABopathies” term that can in turn cover a broad range of manifestations. This study can per se increase the genotype-phenotype spectrum of WARBM type 1.

**Supplementary Information:**

The online version contains supplementary material available at 10.1186/s12883-021-02204-w.

## Background

Warburg Micro syndrome (WARBM)—also known as Micro Syndrome—is a rare autosomal recessive disorder with unknown true incidence. This syndrome is characterized by neurodevelopmental abnormalities such as congenital or postnatal microcephaly, severe intellectual disability, pachy- or polymicrogyria, and hypoplasia/agenesis of the corpus callosum in addition to the ocular manifestations including congenital cataract, microcornea, microphthalmia, and optic atrophy [1, 2]. This syndrome embraces four subtypes as in WARBM type 1 (MIM: 600118) which is caused by biallelic mutations of the RAB3 GTPase-activating protein 1 gene (*RAB3GAP1*; MIM: 602536) [3], WARBM type 2 (MIM: 614225) by mutations of *RAB3GAP2* (MIM: 609275) [4], WARBM type 3 (MIM: 614222) by mutations of the *Ras-related protein Rab-18* (*RAB18*; MIM: 602207) [1], and WARBM type 4 (MIM: 615663) by mutations in the *TBC1 Domain Family Member 20* (*TBC1D20*; MIM: 611663) [5]. Of these, *RAB3GAP1* mutations are by far the most common and approximately account for 40% of WARBM cases [5].

*RAB3GAP1* contains 24 exons and encodes the catalytic subunit of a Rab GTPase activating protein (GAP) that has specificity for the Rab3 subfamily (i.e. RAB3A, RAB3B, RAB3C, and RAB3D) [6]. The catalytic activity of RAB3GAP1 is attributed to its C-terminus (i.e. codons 601–981) [7]. Rab3 proteins are required for normal eye and brain development [8], proper exocytosis of neurotransmitters [9] and hormones [10], and also are imperative for other neurodevelopmental processes such as proliferation, migration, and differentiation of neuronal cells [11, 12]. Rab3A is the most common isoform in the human brain playing a pivotal role in neurotransmitter release and synaptic plasticity of RAB3GAP, which, in turn, converts active Rab3-GTP to inactive Rab3-GDP [13].

One of the major obstacles for clinicians presented with patients with a combination of different symptoms—e.g. postnatal growth retardation, microcephaly, microphthalmia, and cataracts—is to make a differential diagnosis [14]; however, using some advanced technologies such as whole-exome sequencing (WES), to some extent, has addressed these concerns and helped to make as precisely as diagnosis.

Although different studies have been conducted to investigate into the genotype-phenotype correlation in WARBM patients, yet this still remains blanketed in mystery in different populations. To fill this gap, we describe the clinical characteristics of two Iranian patients with WARBM type 1. This study may provide insight into the pathogenesis and clinical spectrum and facilitate early diagnosis of this syndrome.

## Case presentation

Two patients—a 5-year-old female and a 4.5-year-old male—were referred to our department due to postnatal microcephaly and ocular manifestations including congenital cataract, microphthalmia, and optic atrophy. Pregnancies were uneventful; the anthropometric data (e.g. weight, length, and head circumference) in their first month of life were reported normal. No contractures were detected in both patients, while mild facial hypertrichosis was evident and limited to the temporal areas of their faces. Their parents were phenotypically normal and also consanguineous. The mothers (II.1 and II.3) had not any confrontation with infection, radiation, or even drug consumption during their pregnancies.

All of the patients’ clinical information and medical history were collected at the Department of Medical Genetics, Tarbiat Modares University, Tehran, Iran. Detailed patients’ history including disease onset, symptoms, progression, and also family history were gathered at this department. In addition to the traditional biochemistry tests, the patients were subjected to karyotyping using standard procedures to exclude any detectable chromosomal abnormalities. Neuroimaging was carried out using non-contrast brain magnetic resonance imaging (MRI).

### Patient 1

The proband (III.1), a 5-year-old female, was born through normal vaginal delivery at 34 weeks of gestation without any complications. Her birth weight, length, and head circumference (HC) were reported normal. The motor developmental delay was firstly noted after the age of 6 months. After this age, the patient was not able to roll over or even sit on her own. On top of that, she had a severe speech and cognition delay. No seizure was reported by the parents until examination time. Cataract surgery was performed on the patient at the age of 4 years.

**Fig. 1:**
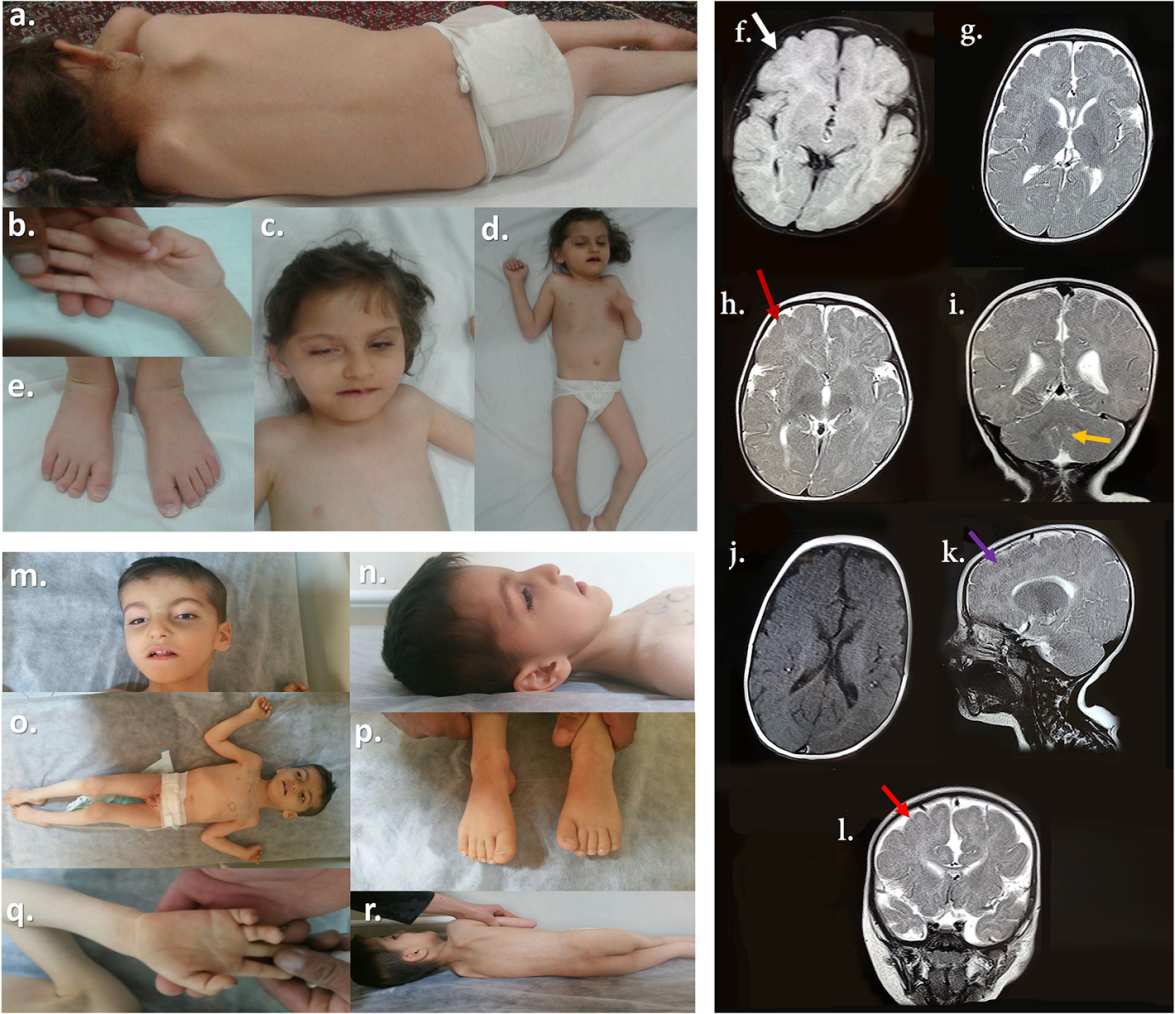
A summary of important patients’ clinical features. The proband (III.1) shows mild thoracolumbar scoliosis and hypertrichosis of the upper back (in the cervical region) (**a**), the cortical thumb and no contractures (**b**), mild unilateral ptosis of the right eye and low anterior hairline limited to the temporal sides (**c, d**), and normal feet fingers and toes (**e**). Selected brain MRI sequences of the index case (III.1) at the age of 5 years: Axial T1-W and coronal T2-W images reveal pachygyria in the frontal area (white arrow) and widened Sylvian fissures (**f**), axial and coronal T2-W images demonstrate diffuse abnormal signal changes in deep and subcortical white matter areas and cortical thickening especially in frontal area (**g-i**; red and yellow arrows), axial T1-W image illustrates a hypomyelination pattern of deep white matter with partial myelination of LLIC (Lower Limb of Internal Capsule) (**j**), sagittal T2-weighted MRI scan casts light on enlarged ventricles and agenesis of the corpus callosum and subcortical band heterotopia (**k**; purple arrow), coronal T2-weighted shows hypomyelination in the pre-dentate white matter of the cerebellum (**l**; thick red arrowhead)**.** The second patient (III.4) manifested some important clinical features as in downturned corners of the mouth, wide nasal bridge, relatively short nose, mild micrognathia, a mild prominent forehead with bitemporal hollowing (**m**), bilateral low-set prominent ears, low anterior hairline in lateral sides, and prominent antitragus (**n**), limb spasticity and hypogenitalism with micropenis and bilateral cryptorchidism (**o**), overlapping feet toes (**p**), normal fingers and palm (**q**), and mild scoliosis and hypertrichosis of the upper back (**r**)

Physical and neurologic examination at the age of 5 years revealed a severe head lag, axial hypotonia, sparse voluntary movements, and peripheral spasticity in addition to severe speech and intellectual disability. Head circumference was measured 46 ± 0.2 cm (<− 3 SD) showing postnatal progressive microcephaly. From skeletal points of view, mild thoracolumbar scoliosis (Fig. 1a) and cortical thumb (Fig. 1b) were evident. Some craniofacial features—e.g. large low-set ears, congenital bilateral cataract, microphthalmia, and ptosis (Fig. 1b, c, d**—**were also apparent. No obvious abnormality was found regarding the patient’s feet (Fig. 1e).

Brain MRI without contrast including T1-W, T2-W, and FLAIR sequences was performed on the proband at the age of 5 years and revealed agenesis of the corpus callosum, cerebral white matter hypomyelination as diffuse bilateral abnormal signal changes in subcortical, deep, and periventricular white matter areas, cortical thickening especially in the frontal area, subcortical band heterotopia in the anterior part of the brain, and hypomyelination in the pre-dentate white matter of the cerebellum (Fig. 1f-l). Similar findings were obtained for the III.4. Further examinations showed no other abnormality using echocardiography, abdominal ultrasonography, and further skeletal investigations—i.e. examination for osteopetrosis, kyphoscoliosis, osteopenia, and growth hormone deficiency. Besides, using auditory brainstem response (ABR) and pure tone otoacoustic emissions (OAEs) testing, no hearing impairment was detected in the patient.

Hematological examination, thyroid, liver, and renal function tests, serum calcium, ammonia, lactate, pyruvate, and TORCH studies (serum anti-toxoplasmosis, rubella, cytomegalovirus, and herpes virus IgG and IgM antibodies) were all reported normal. Karyotyping did not reveal any detectable chromosomal abnormalities (Additional file 1). In Table 1, we summarized some of the important clinical findings in this patient, compared to different types of WARBM disorder and Martsolf syndrome (MIM: 212720).

**Table 1:** summary of the important clinical features of the patients. All clinical features are compared to the different types of Warburg Micro Syndrome (WARBM) and Martsolf syndrome

Group	Subgroup	Clinical Feature	III.1	III.4	WARBM1	WARBM2	WARBM3	WARBM4	Martsolf syndrome
aGe at examination	Age (year)	–	5	4.5	**–**	**–**	**–**	**–**	**–**
Intellectual disability^a^	–	–	STP	STP	STP	S	STP	S	MTS
Growth	Height	Short stature	NR	NR	**+**	**+**	**+**	**+**	**+**
		Height less than 5th percentile	–	–	**+**	**+**	**+**	**+**	**+**
		postnatal growth retardation	+	+	**+**	**+**	**+**	**+**	**–**
	Weight	abnormal Weight	–	–	**–**	**–**	**–**	**–**	**+**
Head & neck	Head	Brachycephaly	–	–	**–**	**+**	**+**	**+**	**+**
		Microcephaly	+	+	**+**	**+**	**+**	**+**	**+**
	Face	Micrognathia	+	+	**+**	**–**	**+**	**–**	**+**
		Abnormal philtrum	+	+	**–**	**–**	**+**	**–**	**+**
	Ears	Prominent antitragus	+	+	**–**	**–**	**–**	**–**	**+**
		Posteriorly rotated ears	–	–	**–**	**–**	**–**	**–**	**+**
		Large ears	+	+	**+**	**+**	**+**	**+**	**–**
	Eyes	Cataracts	+	+	**+**	**+**	**+**	**+**	**+**
		Epicanthal folds	–	–	**–**	**–**	**–**	**–**	**+**
		Downslanting palpebral fissures	–	–	**–**	**–**	**–**	**–**	**+**
		Microphthalmia	+	+	**+**	**+**	**+**	**+**	**–**
		Microcornea	NR	NR	**+**	**+**	**+**	**+**	**–**
		Optic atrophy	+	+	**+**	**+**	**–**	**–**	**–**
		Ptosis	+	+	**+**	**–**	**–**	**+**	**–**
		Deep-set eyes	–	–	**+**	**–**	**–**	**+**	**–**
		Atonic pupils	–	–	**–**	**+**	**+**	**–**	**–**
		Nystagmus	**–**	**–**	**–**	**–**	**+**	**–**	**–**
	Nose	Short-nose	+	+	**–**	**+**	**+**	**–**	**–**
		prominent nasal root	+	+	**–**	**+**	**+**	**+**	**–**
		Wide nasal bridge	–	+	**–**	**–**	**–**	**+**	**–**
	Mouth	High palate	–	–	**–**	**–**	**–**	**–**	**+**
		Downturned corners of the mouth	–	+	**–**	**–**	**+**	**–**	**–**
		soft cleft palate	–	+	**+**	**–**	**–**	**–**	**–**
	Teeth	Malaligned teeth	–	+	**–**	**–**	**–**	**–**	**+**
		Prominent secondary alveolar ridges	–	–	**–**	**–**	**+**	**–**	**–**
Cardiovascular	Heart	Cardiomyopathy	–	–	**–**	**–**	**–**	**–**	**+**
		Cardiac Failure	–	–	**–**	**–**	**–**	**–**	**+**
Respiratory	Infection	Recurrent respiratory infections	–	–	**–**	**–**	**–**	**–**	**+**
	Airway	Tracheomalacia	–	–	**–**	**–**	**–**	**–**	**+**
Chest	Breasts	Prominent nipples	–	–	**–**	**–**	**–**	**–**	**+**
		Widely spaced nipples	–	–	**–**	**–**	**+**	**–**	**–**
	Ribs	Pectus carinatum/ excavatum	**–**	**–**	**–**	**–**	**–**	**–**	**+**
Abdomen	Gastrointestinal	Feeding problems	NR	NR	**–**	**–**	**–**	**–**	**+**
Genitourinary	Male	Small penis	ND	+	**+**	**–**	**–**	**–**	**+**
		Hypogenitalism	+	+	**+**	**+**	**+**	**+**	**–**
		Scrotal hypoplasia/ Small testes	ND	+	**–**	**+**	**+**	**–**	**–**
		Cryptorchidism	ND	+	**+**	**–**	**–**	**+**	**+**
	Female	Hypoplasia of labia majora	+	–	**–**	**+**	**+**	**–**	**–**
Skeletal	Limbs	Contractures	–	–	**–**	**+**	**+**	**+**	**–**
		Distal limb contractures	–	–	**–**	**–**	**+**	**–**	**–**
		Joint hypermobility	–	–	**+**	**–**	**–**	**–**	**–**
	Hands	Finger joint laxity	–	–	**–**	**–**	**–**	**–**	**+**
		Joint hypermobility	–	–	**+**	**–**	**–**	**–**	**–**
		Cortical thumbs	+	–	**–**	**–**	**+**	**–**	**–**
	Feet	Deformities of metatarsal bones	–	–	**+**	**–**	**–**	**–**	**–**
		Overlapping toes	+	+	**+**	**+**	**–**	**–**	**–**
Skin, nails, & hair	Nails	Abnormal nails	–	–	**–**	**–**	**–**	**–**	**+**
	Hair	Facial hypertrichosis	+	+	**+**	**–**	**–**	**–**	**–**
		Low anterior hairline	+	+	**–**	**+**	**+**	**+**	**–**
		Hypertrichosis of upper back	+	+	**–**	**–**	**+**	**–**	**–**
Neurologic	Central and peripheral Nervous Systems	Severe intellectual disability	+	+	**+**	**+**	**+**	**+**	**+**
		Truncal hypotonia	NR	NR	**–**	**–**	**+**	**–**	**–**
		Hyperreflexia	NR	NR	**+**	**–**	**–**	**–**	**–**
		Limbs hypotonia	+	+	**+**	**–**	**–**	**–**	**–**
		Axial hypotonia	+	+	**–**	**+**	**–**	**+**	**–**
		White matter signal changes	+	+	**–**	**–**	**–**	**–**	**–**
		Spastic diplegia to quadriplegia	+	+	**–**	**+**	**+**	**+**	**–**
		Increased deep tendon responses	–	–	**–**	**–**	**+**	**–**	**–**
		Ankle clonus	–	–	**–**	**–**	**+**	**–**	**–**
	Neuroimaging Findings	Cerebral malformations	+	+	**+**	**–**	**–**	**+**	**–**
		Hypoplasia of the corpus callosum	–	+	**+**	**+**	**+**	**+**	**–**
		Agenesis of the corpus callosum	+	–	**+**	**+**	**–**	**–**	**–**
		Cerebellar hypoplasia	+	+	**+**	**–**	**–**	**+**	**–**
		Wide Sylvian fissures	+	+	**–**	**+**	**–**	**–**	**–**
		Increased subdural space	+	+	**–**	**+**	**–**	**–**	**–**
		Enlarged ventricles	+	+	**–**	**–**	**+**	**+**	**–**
		Pachygyria	+	+	**+**	**–**	**–**	**–**	
		Polymicrogyria	–	–	**–**	**+**	**+**	**+**	**–**
		Mega cisterna Magna	–	–	**–**	**–**	**–**	**+**	**–**

*Abbreviations*: *STP* severe-to-profound intellectual disability, *S* severe, *MTS* moderate-to-severe intellectual disability, *NR* Not Reported or detected at the age of examination, *ND* Not defined or not-appropriate value for the specific gender

^a^The intelligence quotient (IQ) scores were used to classified the intellectual disability (ID) into mild (IQ 50/55 to 70), moderate (35/40 to 50/55), severe (20/25 to 35/40), and profound (< 25)

### Patient 2

The second case (III.4) was a 4.5-year-old male born through a normal vaginal delivery without any complications. He had normal birth weight and length and his birth HC was measured as 31 ± 0.2 cm (7th centile). No seizures were recorded in the patient’s medical history. According to the medical history, the motor developmental delay was first suspected after the age of 8 months. After this period, the patient was not able to roll over or sit down without any assistant. Furthermore, speech delay and cognitive disability were also noted after 1 year of age. Until the age of 4.5 years, no serious medical evaluation had been performed for a better diagnosis.

Clinical examinations at the age of 4.5 years showed quite similar findings in this patient such as noticeably delayed gross motor development with no head control, axial hypotonia, profound intellectual and speech abilities, and four limb spasticity. HC was measured 49 ± 0.2 cm (<− 3 SD) at the age of examination, indicating postnatal microcephaly. The general examination demonstrated a soft cleft palate, downturned corners of the mouth, wide nasal bridge, relatively short nose, mild micrognathia, a mild prominent forehead with bitemporal hollowing, bilateral low-set prominent ears, hypogenitalism with micropenis and bilateral cryptorchidism, and mild scoliosis (Fig. 1m-q). As a prominent clinical feature, the patient showed hypertrichosis of the upper back (Fig. 1r). Ocular abnormalities such as cataract, nasolacrimal duct obstruction, and bilateral microphthalmia were also detected at the time of examination.

Brain MRI without contrast—including T1-W, T2-W, and FLAIR sequences—performed at this age and showed the hypoplasia of the corpus callosum, cerebral white matter hypomyelination, cortical thickening (especially in the frontoparietal area), widened Sylvain fissure, enlarged ventricles, and hypomyelination in the white matter of the cerebellum. Electrocardiogram, bone scan, neonatal TORCH studies, and basic metabolic panel tests were all normal. G-banded chromosome analysis revealed a normal male karyotype as well (Additional file 1).

### Genetic findings and determination of allele frequency of the candidate variants

**Fig. 2:**
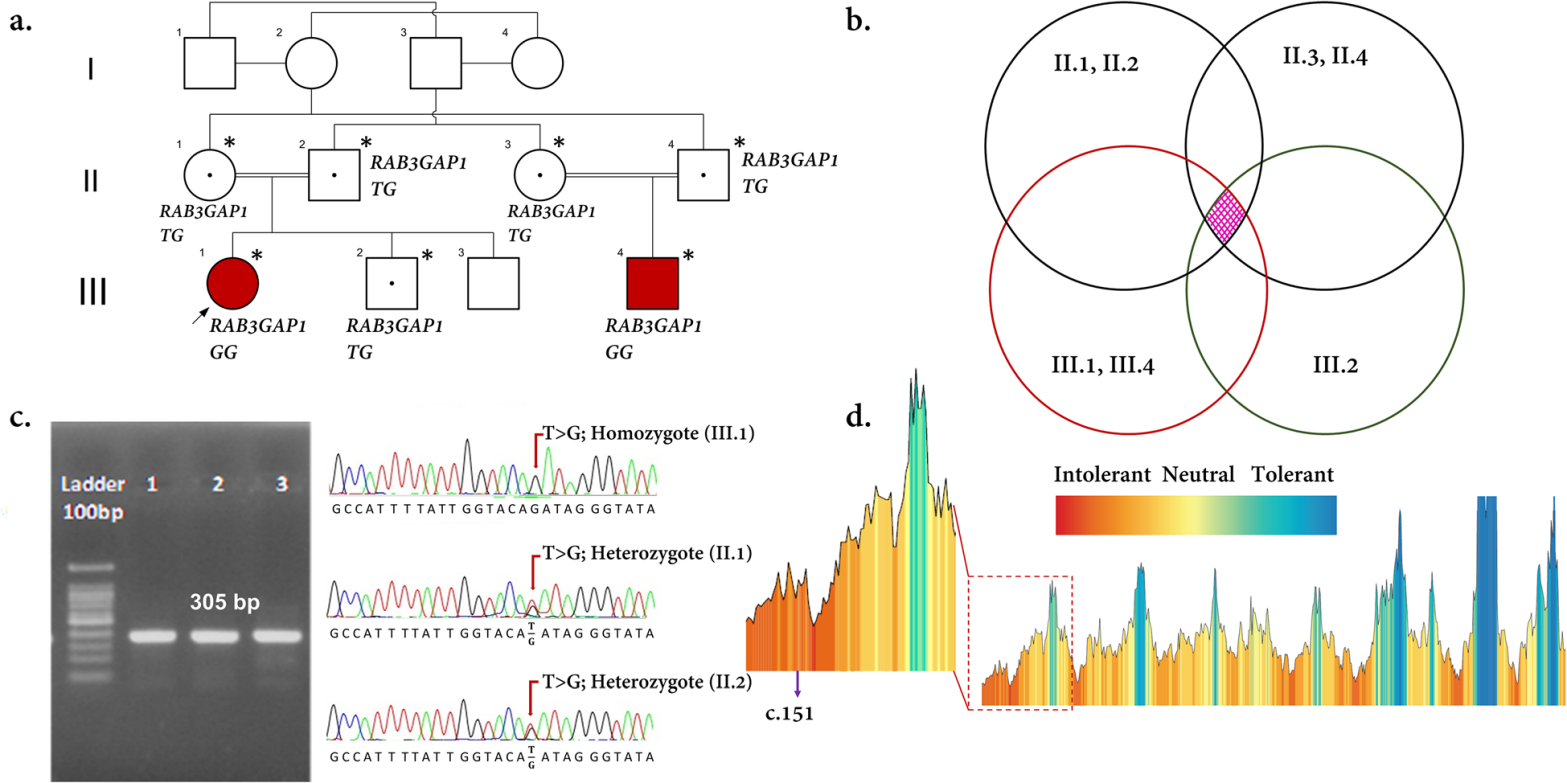
c.151-5 T > G was identified using whole-exome and Sanger sequencing in the family. **a** Pedigree of the family in which the carriers and patients are indicated as TG and GG, respectively. The proband (III.1), III.4, parents (II.1, II.2, II.3, and II.4), and III.2 were subjected to WES (all are shown by an asterisk (*)). In this pedigree, white symbols: unaffected who were homozygous for wild-type allele; red symbol: affected and homozygous for the variant; squares: males; circles: females; parallel lines: consanguineous marriage. The status of *RAB3GAP1* variation is shown for each participant, in which, TG: heterozygote for novel variant and wild-type allele, and GG: homozygote for the variant. **b** Overlapped filtering strategy. The red zone indicates the remained variants for further analysis that are presented as a homozygote in 2 affected members (III.1 and III.4) but not in other individuals. These variants have been seen in heterozygous in other individuals (II.1, II.2, II.3, II.4, and III.2). The purple zone shows the common variants between all individuals. **c** Chromatograms showing nucleotide sequences of c.151-5 T > G of *RAB3GAP1*. In this figure, the size of the sequenced DNA region is shown using 2% gel electrophoresis, in which 1 denotes the patient sample, 2 and 3 show the mother and father samples, respectively. **d** MetaDome was used to identify the intolerant regions (surrounding the c.151-5 T > G variant) in RAB3GAP1. As depicted, the affected nucleotide/residue is located in a highly intolerant region

Due to the heterogeneity of neurodevelopmental disorders [15], we subjected the patients, parents, and also a healthy sibling to paired-end WES screening to detect the possible underlying genetic factor. WES was performed based on the previous studies [16–18]. The mean depth of coverage was around 100× and approximately 98% of targeted regions were covered (Additional file 2). The overlapped filtering strategy was used to detect the common variants among the patients and the parents (Fig. 2a, b). The filtering steps were carried out according to the previous studies [19]. In total, 780,385 common SNV and Indel variants among all the 7 genotyped individuals (are shown by an asterisk (*) in Fig. 2a) were detected. By doing the overlapped filter strategy, 4297 variants were detected. By excluding the synonymous, low-functional profile, and variants with mutation allele frequencies (MAF) greater than 1% in publicly available databases—e.g dbSNP150 [20], 1000 Genomes Project [21], Exome Sequencing Project [22], and gnomAD [23] or ExAC databases [24]—only three variants were identified in *MAP 3 K19, RAB3GAP1*, and *XIRP2* genes, respectively. Different tools, e.g. SIFT [25], Polyphen-2 [26], MutationTaster [27], and Provean [28] were also executed to evaluate the pathogenicity of the identified variants (Table 2). To investigate the co-segregation analysis, the primers were designed using Primer3.0 online tool [29] (Additional file 3) and the variants were subjected to Sanger sequencing (Additional file 4; Fig. 2c). The reference sequences of NM_001199144.1 for *XIRP2,* NM_001018044.2 for *MAP3K19*, and NM_012233.2 for *RAB3GAP1* were used based on the human genome assembly GRCh37 (hg19).

**Table 2:** The pathogenicity prediction of the candidate variants is summarized in this table. The annotation is based on Human GRCh37/hg19

Gene	NM	Ref/Alt	Chr. Position	Alternation	dbSNP	MutationTaster	SIFT	Provean	HSF	Polyphen-2	gnomAD	1000 Genome	Iranome
*XIRP2*	NM_001199144.1	T/C	Chr2:168107737	c.9835 T > C; p.(Ser3279Pro)	rs199990336	B	B	B	–	PD	1.93e-3	NR	0.01063
*MAP3K19*	NM_001018044.2	T/C	Chr2:135745772	c.332 T > C; p.(Ile111Thr)	–	B	PD	B	–	B	NR	NR	NR
*RAB3GAP1*	NM_012233.2	T/G	Chr2:135848563	c.151-5 T > G; p.(Gly51IlefsTer15)	–	D	–	–	D	–	NR	NR	NR

*Abbreviations*: *B* Benign, *D* Damaging, *PD* Probably Damaging, *NR* Not-reported

In the nucleotide level, conservational analyses using ConSurf [30] and ‘2-Way Pseudogene Annotation Set’ of UCSC [31] showed that c.151 T is located in a highly conserved region (Fig. 2d). On the other hand, c.9835 T > C, p.(Ser3279Pro) in *XIRP2* and c.332 T > C, p.(Ile111Thr) in *MAP3K19* were categorized as non-conserved variants. MetaDome [32] was also used to visualize and confirm the data (Additional file 4).

To exclude the variants with low detrimental effects, we determined the allele frequency in 300 healthy and ethnically matched controls. For this purpose, we used PCR-Restriction Fragment Length Polymorphism (PCR-RFLP) and also tetra-primer Amplification Refractory Mutation System-PCR (tetra-primer ARMS-PCR). Using PCR-RFLP (Additional file 2), the allele frequency of c.9835 T > C variant in *XIRP2* was determined 0.031, moreover, a homozygous individual was detected in healthy controls (Additional file 5). c.9835 T > C variant causes a loss of the XapI restriction site. The PCR products were digested with XapI and run on 2% agarose gel to assess whether the variant allele existed or not. This data was in line with the findings of Iranome [33] (allele frequency: 0.01063), suggesting that this variant cannot be considered pathogenic, so we excluded the variant from further investigations.

**Fig. 3:**
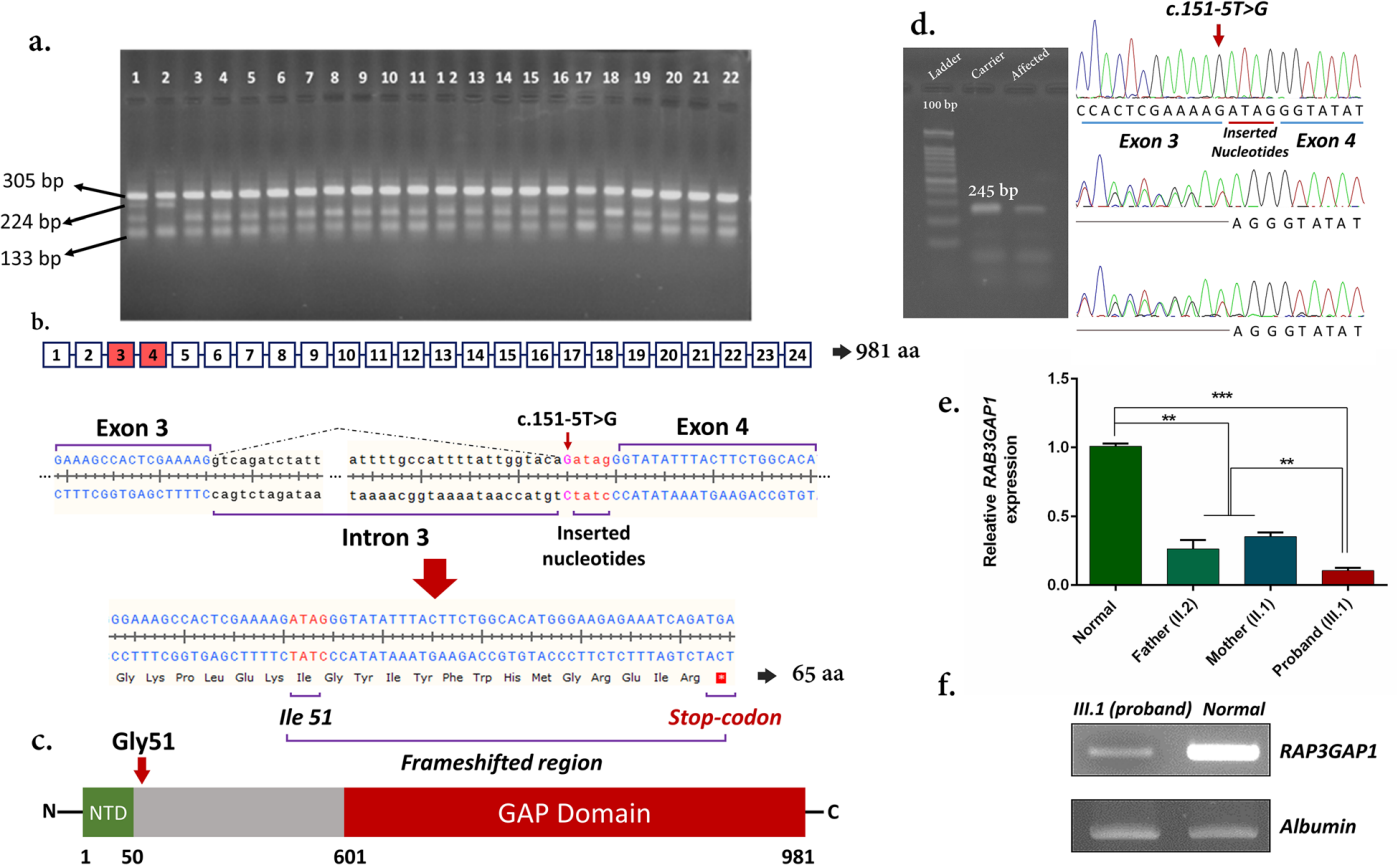
Determining the allele frequency of c.151-5 T > G variant and expression of *RAB3GAP1*. **a** a tetra-primer ARMS-PCR was used to determine the allele frequency of c.151-5 T > G variant on the *RAB3GAP1* gene. In this figure, well 1: heterozygous for wild-type allele, well 2: Homozygous for c.151-5 T > G variant, well 3: homozygous for the wild-type allele. **b** a schematic figure shows the 24 exons of *RAB3GAP1* and also the effects of c.151-5 T > G on splicing. The c.151-5 T > G inserts 4 nucleotides that in turn cause frameshifting and generating the truncated protein. **c** Schematic of the RAB3GAP1 protein showing the Rab3 GAP domain (red) and the conserved N-terminal region of RAB3GAP1 (NTD; green). **d** RT-PCR was performed on RNA samples of patients and carriers (Parents). Chromatograms revealing nucleotide sequences of c.151-5 T > G of *RAB3GAP1*. In this figure, the size of the sequenced cDNA region is shown using 2% gel electrophoresis. **e** and **f** Relative expression levels of *RAB3GAP1* in peripheral blood cells of the patient (proband), parents, and normal individual (III.3) shows a significant reduction in the patient’s mRNA level. III.3 prescreened for c.151-5 T > G variant. The experiment was performed in triplicate and *ALB* (albumin) was used as an internal reference gene. The data are presented as the mean ± standard deviation (SD) and the statistical significance was evaluated by Student’s t-test. Statistical analysis was performed by SPSS version 24.0 (SPSS Inc., Chicago, IL, USA) and GraphPad Prism v.8.0 (GraphPad, San Diego, USA). *P*- values < 0.05 were considered to be statistically significant. ** *P* < 0.01, *** *P* < 0.001

To determine the allele frequency of c.332 T > C in *MAP3K19* and c.151-5 T > G in *RAG3GAP1* in 300 healthy ethnicity-matched controls, tetra-primer ARMS-PCR was performed **(**Additional File 6; Fig. 3a). No homozygote/heterozygote was identified among 300 samples; consistently, Iranome and gnomAD verified these findings. The primers and size of expected bands in each reaction are depicted in Additional file 3. The procedure was carried out according to the previous study [34] (Additional file 2).

### c.151-5T> G affects splicing

Using ‘MaxEnt Acceptor site algorithms’ of Human Splicing Finder V.3.1 (HSF; https://hsf.genomnis.com/) [35], we showed that c.151-5 T > G may alter the wild-type acceptor site, thus can most probably affect splicing. This finding was confirmed by Berkeley Drosophila Genome Project (BDGP; https://www.fruitfly.org) [36] and NetGene2 (http://www.cbs.dtu.dk/services/NetGene2) [36] tools. To predict if the variant can affect the cryptic splice-site activation and exon skipping, CRYP-SKIP (https://cryp-skip.img.cas.cz/) program [36] was used; these data showed that the c.151-5 T > G variant can increase the probability of cryptic splice site activation. To confirm, we used RT-PCR on the samples of the proband (III.1) and her parents (II.1 and II.2). The PCR products were then sequenced using Sanger sequencing with same primers (Additional file 3). The results revealed that the novel variant can activate a cryptic splice site within intron 3 of *RAB3GAP1*. This can change the splicing process of the *RAB3GAP1* gene by adding 4 nucleotides between exons 3 and 4. In sum, this leads to the losing 930 amino acids (Fig. 3b-d).

The relative expression levels of *RAB3GAP1* in the peripheral blood cells of the index case (III.1), parents (II.1 and II.2), and also a control/normal individual (III.3) were compared. The normal individual (control) was initially screened for the c.151-5 T > G variant and he was negative for this variant. RT-qPCR was performed using SYBR® Premix Taq™ II (TAKARA, Tokyo, Japan) on an ABI StepOne Sequence Detection System (Applied Biosystems, Foster City, USA). Each experiment was performed in triplicate and expression data were normalized to *ALB* (albumin) as an internal reference gene. Primer sequences are put forward in Additional file 3. The 2^−ΔΔCt^ method was used for quantification by comparing the Ct values [37, 38]. The RT-qPCR analysis showed that the *RAB3GAP1* expression was significantly decreased in the proband (Fig. 3e, f) and also demonstrated that only a trivial amount of *RAB3GAP1* expression remained as a result of homozygous c.151-5 T > G splice site variant. This underscores the potential pathogenic effect of this novel variant (Fig. 3b, c). On the other hand, the decreased expression of *RAB3GAP1* was determined in parents who were heterozygous for the novel variant (Fig. 3e, f).

To show whether the impaired genes make a contribution to brain development/function, we explored the regional expression of *MAP3K19* and *RAB3GAP1* in the normal adult human brain. For this purpose, we used microarray data (Affymetrix Exon 1.0 ST) from human post-mortem brain tissue collected by the UK Human Brain Expression Consortium [39]. To confirm, we also used the data available in the Allen Mouse Brain Atlas [40, 41]. To show how the expression of *Map3k19* (MGI: 1203481) and *Rab3gap1* (MGI: 2445001) changes during brain development, we utilized the ‘Gene Expression Database (GXD) Project’ [42] of MGI which is an international database resource for the laboratory mouse. The results showed that *Map3k19* has a subtle expression during brain development that per se cannot justify a broad range of brain abnormalities in the patients. On the other hand, the aberrant expression of the *RAB3GAP1* was significantly expected to be associated with the diverse brain impairments in WARBM disorders (Additional file 7).

## Discussion and conclusions

WARBM is a heterogeneous group of autosomal recessive disorders that can be resulted from loss-of-function mutations in *RAB3GAP1/2*, *RAB18*, and *TBC1D20* [43, 44]*.* These genes encode the ‘RAB proteins’ that mainly function as molecular switches [45]; they can also regulate membrane trafficking in a spatially and temporally restricted manner [46]. GAP proteins can regulate RAB protein cycling [47], e.g., RAB3GAP1 (catalytic subunit) and RAB3GAP2 (non-catalytic subunit) make a heterodimeric enzyme complex, which has GAP activity that is specific for the RAB3 family of protein. This heterodimeric enzyme complex hydrolyzes GTP into GDP and also modulates the Ca^2+^ mediated exocytosis of hormones and neurotransmitters [48].

Most of the mutations in *RAB3GAP1—*in WARBM patients—are predicted to result in stop-codons and are distributed throughout the coding region from codons 89 to 934 [3]. In a nutshell, all kinds of these mutations can lead to a truncated protein either before or within the regions that are important for catalytic activity [3] (Fig. 3c**)**. This also can explain why there are some phenotypic variations between the WARBM with different mutations in *RAB3GAP1*. *Rab3gap1* mutant and knockout mice models have previously been generated, while none of them embraced all major clinical features of WARBM [49]. In mice, disruption of *Rab3gap1* results in increased short-term depression and also paired-pulse facilitation at CA1 hippocampal neurons [49]. On the other hand, human case reports have successfully covered a broad range of phenotypes caused by mutations in *RAB3GAP1*.

The majority of WARBM cases, similar to our case, are resulted from consanguineous marriages [50]. Here, after performing WES, as a final candidate, we identified a homozygous pathogenic variant c.151-5 T > G; p.(Gly51IlefsTer15) in the intron 3 of the *RAB3GAP1* gene, justifying the phenotypes that were in association with autosomal recessive WARBM type 1. Data from in silico analyses and also RT-PCR showed that c.151-5 T > G can activate a cryptic splice site in intron 3. This led to the addition of four nucleotides upstream of exon 4, which in turn changes the mRNA reading frame; this causes a loss of 971 codons, due to the premature stop codon. Moreover, RT-qPCR data showed that the expression of *RAB3GAP1* harboring c.151-5 T > G variant was significantly decreased in the patients than the normal individual (III.3). Although the parents showed lower mRNA levels than the control group, they did not show any symptoms of the disease. This can per se confirm that the low amount of RAB3GAP1 protein is also sufficiently functional. There are two fates for mRNAs containing premature termination codons: nonsense-mediated mRNA decay (NMD) [51] or translation to truncated proteins [52]. NMD is an evolutionarily conserved quality control pathway in eukaryotic cells that is responsible for inspecting mRNA for any possible errors, so eliminating any error-containing transcripts and controlling the amount of non-mutated transcript in the transcriptome (reviewed in [53]). Therefore, NMD leads to loss-of-function allele [51]; herein, RT-qPCR data supported the NMD hypothesis. Axiomatically, RAB3GAP1 protein that embraces p.(Gly51IlefsTer15) will be destroyed or be malfunctioned. It was previously suggested that pathogenic mutations in *RAB3GAP1* with WARBM type 1 were mostly frameshift, nonsense, and splicing mutations causing complete loss of function [54]; Our data underscore this hypothesis as well. We also reclassified the variant based on the American College of Medical Genetics and Genomics (ACMG) variant interpretation guideline [55] into the ‘*Pathogenic*’ variant.

Homozygous and compound heterozygous mutations in human *RAB3GAP1* cause WARBM type 1 and also a somehow milder phenotype called Martsolf syndrome [6]. These two disorders are genetically heterogeneous and comprise a phenotypic spectrum described as “RAB18 deficiency” based on molecular etiology [6]. Mutations in *RAB3GAP2* can cause Martsolf syndrome. To exclude this syndrome, we meticulously compared the phenotypes of the patients in Table 1. Indeed, patients with Martsolf syndrome manifest different degrees of ID—from mild to severe—while WARBM patients show often severe-to-profound ID with no or even very limited communication skills. Spastic quadriplegia has been reported in WARBM, whereas in Martsolf syndrome, spasticity is limited to lower limbs [56]. Molecularly, mutations reported in association with WARBM are mostly predicted to result in NMD and/or loss-of-protein-function [57]. On top of that, we also found that some clinical features, e.g. hypoplasia of the corpus callosum, microcephaly, and developmental delay, can be observed in other types of WARBM in addition to Martsolf syndrome. Besides, based on some common clinical features among these patients and Martsolf syndrome, e.g. prominent antitragus, malaligned/misaligned teeth, and small penis, we suggest using the “RABopathies” term that can efficiently cover all clinical features between different types of WARBM and Martsolf syndrome.

We also introduced some novel manifestations of WARBM type 1 including abnormal philtrum, prominent antitragus, downturned corners of the mouth, malaligned teeth, scrotal hypoplasia, low anterior hairline in temporal areas, hypertrichosis of upper back, spastic diplegia to quadriplegia, and signal changes in deep and subcortical white matter areas. Perhaps, one of the most recognizable features of WARBM is the brain phenotype. Herein, clinical examinations showed that the patients (III.1 and III.4) manifested postnatal microcephaly which has been observed in most cases of the Martsolf syndrome and the WARBM. Developmental milestones in two patients were compatible with severe ID (Table 1).

The present study demonstrated that a novel homozygous variant in *RAB3GAP1* is the main cause of WARBM type 1 disorder characterized by abnormal philtrum, prominent antitragus, downturned corners of the mouth, cerebral white matter signal changes, malaligned teeth, scrotal hypoplasia, low anterior hairline, hypertrichosis of upper back, spastic diplegia to quadriplegia as novel manifestations in WARBM type 1. We also coined the term “RABopathies” that can cover the overlapped clinical features among WARBM and Martsolf syndrome. Moreover, we reconfirmed that the WES can properly detect underlying genetic factors contributing to the neurodevelopmental disorder that show a great phenotype overlapping. This study also provides important information to guide genetic counseling and expands the genotype-phenotype spectrum of WARBM type 1.

## Supplementary Information

**Additional file 1.** The karyotypes of the proband (a) and the male affected individual or III.4 (b). The karyotypes did not show any obvious chromosomal changes

**Additional file 2.** Detailed procedures of Whole-Exome Sequencing and Tetraplex-Amplification refractory mutation system (Tetraple-ARMS-PCR)

**Additional file 3.** A list of primers that was used in this study

**Additional file 4. **Sanger sequencing and conservational analysis for the candidate variants in *MAP3K19* and *XIRP2* genes. **a)** Chromatograms shed light on the cosegregation of c.332 T > C of *MAP3K19* in the family members. **b)** Chromatograms showing nucleotide sequences of *XIRP2* in the regions of c.9835 T > C. **c)** MetaDome shows that the c.332 T is located in a region with an average conservational profile in MAP3K19 protein. **d)** In this XIRP2 tolerance landscape, the region harboring the novel missense variant is partially tolerant in comparison with other parts in this protein. The affected region is located in a somehow variable region

**Additional file 5. **2% agarose gel electrophoresis of tetra primer ARMS-PCR test products for c.332 T > C in the *MAP3K19* gene. In this figure: well 1: 100 bp ladder, well 2: negative PCR test control (no template control; NTC), well 3: Homozygous for the normal allele, well 4: Heterozygous for the wild-type allele. The allele frequency was estimated at 0.031 for the c.332 T > C variant

**Additional file 6. **A simple and rapid PCR-RFLP assay was used to detect the c.9835 T > C variant in the *XIRP2* gene. **a)** This schematic figure shows the generated fragments after digestion with the XapI restriction enzyme. The variant causes losing the restriction enzyme site. Agarose gel (2.0%) electrophoresis with ethidium bromide staining following the XapI digestion of the PCR products is shown. PCR-RFLP results in normal control showing 340, 249, and 130 bp (T/T: wild-type allele); after XapI digestion, a heterozygous sample will show four distinct bands including 470, 340, 249, and 130 bp. The homozygote reveals three distinct bands consisting of 470, 249, and 130 bp. **b)** PCR-RFLP assay was used on the samples in order to show the genotyping. In this figure, 1: heterozygote, 2: patient, 3–14, and 16–22 are wild-type alleles

**Additional file 7. a)** Gene expression of the *RAB3GAP1* is changing in different brain regions as age increases. **b)** Gene expression of *RAB3GAP1* in different brain regions and sexes. **c)** Boxplots of RAB3GAP1 mRNA expression levels in ten adult brain regions. The expression levels are based on exon array experiments and are plotted on a log2 scale (y-axis). This dataset was generated with Affymetrix Exon 1.0 ST arrays and brain tissue originating from 134 control individuals, collected by the Medical Research Council Sudden Death. The plot also shows significant variation in *RAB3GAP1* expression across the ten brain regions analyzed, such that expression is higher in the temporal cortex than in any other region. In this figure, TCTX: temporal cortex, FCTX: frontal cortex, OCTX: occipital cortex, SNIG: substantia nigra, THAL: thalamus, PUTM: putamen, HIPP: hippocampus, CRBL: cerebellar cortex, MEDU: medulla (specifically the inferior olivary nucleus), WHMT: intralobular white matter, and N: number of samples analyzed for each brain region. These figures shed light on *RAB3GAP1* expression in the different parts of the brain

## Data Availability

The datasets used and/or analyzed during the current study are available from the corresponding author on reasonable request. The variant and pertinent phenotypes caused by a mutation in *RAB3GAP1* are accessible at ClinVar (SCV001426394) and Leiden Open Variation Database (LOVD; individual number: 279559).

## Abbreviations

ACMG: American College of Medical Genetics and Genomics
GXD: Gene Expression Database
HC: Head circumference
MRI: Magnetic resonance imaging
PCR-RFLP: PCR-Restriction Fragment Length Polymorphism
NMD: Nonsense-mediated mRNA decay
RT-PCR: Reverse transcription-PCR
RT-qPCR: Quantitative reverse transcription-PCR
Tetra-primer ARMS-PCR: Tetra-primer Amplification Refractory Mutation System-PCR
WARBM: Warburg micro syndrome
WES: Whole-exome sequencing
