# Supplementary material for: Novel manifestations of Warburg micro syndrome type 1 caused by a new splicing variant of RAB3GAP1: a case report
Source: BMC Neurol. 2021 Apr 28;21:180. doi: 10.1186/s12883-021-02204-w (PMC8080372; doi:10.1186/s12883-021-02204-w)

Additional File 1. The karyotypes of the proband (**a**) and the male affected individual or III.4 (**b**). The karyotypes did not show any obvious chromosomal changes.

a.

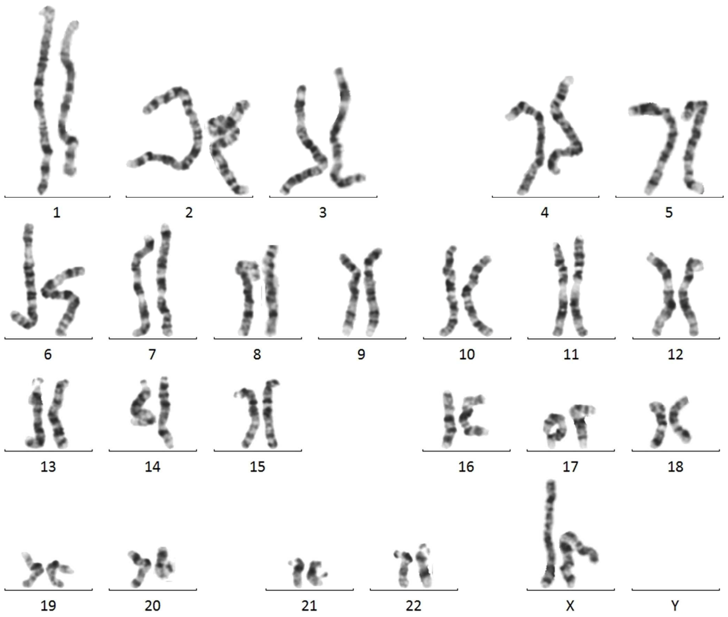

b.

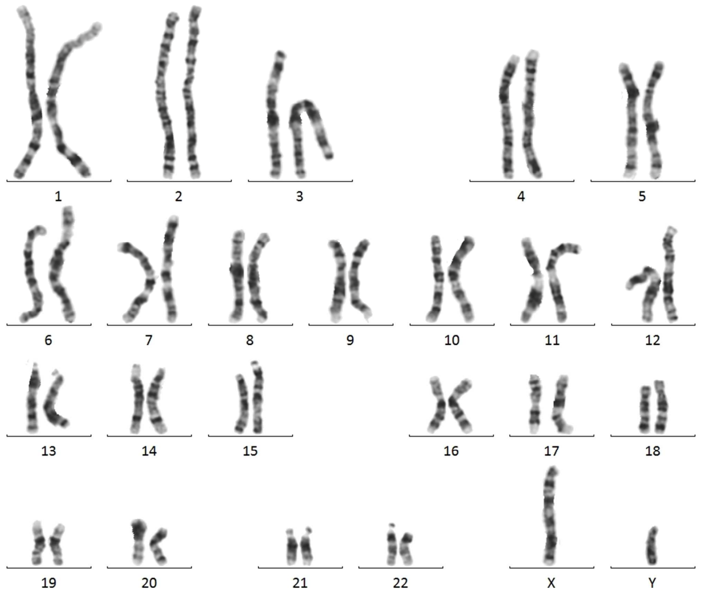

Supplement: Supplementary file 1 — Additional file 1. The karyotypes of the proband (a) and the male affected individual or III.4 (b). The karyotypes did not show any obvious chromosomal changes [file 12883_2021_2204_MOESM1_ESM.pdf]
