# Supplementary material for: Novel manifestations of Warburg micro syndrome type 1 caused by a new splicing variant of RAB3GAP1: a case report
Source: BMC Neurol. 2021 Apr 28;21:180. doi: 10.1186/s12883-021-02204-w (PMC8080372; doi:10.1186/s12883-021-02204-w)
Supplement: Supplementary file 2 — Additional file 2. Detailed procedures of Whole-Exome Sequencing and Tetraplex-Amplification refractory mutation system (Tetraple-ARMS-PCR) [file 12883_2021_2204_MOESM2_ESM.docx]

Novel manifestations of Warburg Micro Syndrome Type 1 caused by a new splicing variant of *RAB3GAP1*

Raziyeh Khalesi ^1^, Ehsan Razmara ^1^, Golareh Asgaritarghi ^2^, Ali Reza Tavasoli ^3^, Yasser Riazalhosseini ^4,5^, Daniel Auld ^4,5*^, Masoud Garshasbi ^1,*^

1. Department of Medical Genetics, Faculty of Medical Sciences, Tarbiat Modares University, Tehran, Iran.
2. Department of Genetics, Faculty of Biological Sciences, Tarbiat Modares University, Tehran, Iran.
3. Myelin Disorders Clinic, Pediatric Neurology Division, Children's Medical Center, Pediatrics Center of Excellence, Tehran University of Medical Sciences, Tehran, Iran.
4. McGill Genome Centre, Montréal, Québec, Canada.
5. Department of Human Genetics, McGill University, Montréal, Québec, Canada

## Additional file 2: detailed procedures of Whole-Exome Sequencing and Tetraplex-Amplification refractory mutation system (Tetraple-ARMS-PCR)

## DNA Extraction

About 10 ml of peripheral blood was collected from each individual and genomic DNA (gDNA) was extracted using with MagPurix kit (ZP02001, Zinexts company, Taiwan).

## Nanodrop DNA quantification

Absorbance‐based quantification of nucleic acid was obtained with Thermo Scientific TM Nanodrop 2000^®^ (Thermo Fisher Scientific, Wilmington, DE, USA) after pipetting 1 μl of each sample onto the end of the fiber optic cable. Duplicate analyses were performed on each sample, setting 0.2 mm path length to calculate the absorbance, and the results were generated in ng/μl. Then the two values obtained for each sample were averaged. The ratio of sample absorbance at 260 nm and 280 nm was used to assess the purity of DNA.

**Full methods of the whole-exome sequencing (WES)**

***1. Construction of pre-enrichment DNA libraries***

Pre-enrichment DNA libraries were constructed using the Illumina TrueSeq DNA sample preparation kit (Illumina, Inc., San Diego, CA, USA). For this purpose, the genomic DNA (gDNA) was isolated, and the qualified gDNA sample was randomly fragmented into the fragments were between 200 and 300 bp. Thence, adapters were ligated to both ends of the fragments and the adapter-ligated templates were purified using AMPure XP beads (Beckman **Coulter, Inc.,** Brea, CA, USA), and fragments with an insert size of approximately 300–400 bp were excised.

***2. Exome enrichment***

Approximately a total of 0.5 µg of each library was pooled. DNA was hybridized overnight using Illumina TrueSeq Exome Enrichment probes (Illumina, Inc.). Then, the biotinylated probes and the hybridized sample DNA were captured via streptavidin beads and rinsed in a three-step process, and then DNA was eluted from the probes. The above processes were repeated one more time on eluted DNA. Successfully captured DNA fragments containing ligated adapter sequences were enhanced via PCR using adapter-specific primers. The DNA was then isolated using AMPure XP beads and analyzed using the Agilent Bioanalyzer 2100 (Agilent Technologies, Inc., Santa Clara, CA, USA).

***3. Cluster generation of the enriched exome libraries***

The enriched exome libraries were loaded onto flow cells of an Illumina cBot for cluster generation using a TrueSeq PE Cluster Kit v3-cBot-HS (Illumina, Inc.). One lane of each flow cell was reserved for a PhiX control.

***4. Exome sequencing***

The flow cells with the clusters of the enriched exome libraries were then transferred to HiSeq2000 (Illumina, Inc.). High-throughput sequencing was performed for each captured library to ensure that each sample met the desired average sequencing depth of at least 40×.

***5. Alignment of sequences***

Raw reads in FASTQ format from the exome sequencing were aligned to the hg19 reference genome downloaded from UCSC with BWA (bwa-0.5.9; BWA is a software package for mapping low-divergent sequences) using a seed length of 45 as the default parameter. The aligned reads were processed, and PCR duplicates were removed with the Sequence Alignment/Map (SAM) format (SAMtools-0.1.16) (<http://samtools.sourceforge.net>) and processed with the Count Covariates, Table Recalibration, Realigner Target Creator, Indel Realigner step with GATK (GenomeAnalysisTK-1.4) (Broad Institute, Inc. Cambridge, MA, USA).

***Detection of single nucleotide variants (SNVs) and insertions or deletions (Indels)***

SNVs and Indels were detected with GATK according to the previous reports [1].

## Bioinformatics Analysis

Raw-sequence data analysis involving the alignment of reads to the GRCh37/hg19 genome assembly, primary filtering of low-quality reads and probable artifacts, base-calling, and subsequent annotation of variants was performed using an end-to-end in-house bioinformatics pipeline. Relevant variants reported in the Human Gene Mutation Database (HGMD^®^) [2] and ClinVar [3], as well as all variants with minor allele frequency (MAF) of less than 1% in the Genome Aggregation Database (gnomAD) [4], were considered. Additionally, medical history and clinical information were used to evaluate the identified variants.

In brief, four major steps were taken to prioritize all variants. After pooling the variants (selecting heterozygotes in parents and III.2 and homozygous variants in patients), the following filtering steps had been taken forward; to begin with, variants within intergenic, intronic, and untranslated regions were carefully excluded. All previously identified SNPs with allele frequencies of 1% or higher in dbSNP150 (<http://www.ncbi.nlm.nih.gov/projects/SNP>), 1000 Genomes Project [5], Exome Sequencing Project (ESP) (<http://evs.gs.washington.edu/EVS)>, and gnomAD were excluded. A conservational analysis was used to determined the most detrimental variants. In closing, the phenotypes of the patients were used to reevaluate identified variants.

For further consideration, the frequency of the variant was checked out on the local database, Iranome [6]. The possible pathogenic effects of the detected variant were evaluated by in-silico predictors such as Polyphen-2 [7], SIFT [8], and MutationTaster [9].

## Sanger sequencing

Primers surrounding the region of the identified variants were designed by using Primer3.0 (<http://bioinfo.ut.ee./primer3-0.4.0>) [10]. Each 50 µl PCR reaction contained 2 U of Platinum Taq DNA Polymerase (Invitrogen; Thermo Fisher Scientific), 5 µl of PCR buffer (10×), 50 mM of MgCl2 (final 1.5 mM), 10 mM dNTPs mix (final 0.2 mM), 0.2 µM of each primer, and 150 ng of genomic DNA template. The mixture was denatured at 94°C for 2 min and the PCR was performed for 35 cycles in a thermocycler (GenePro TC‐E‐48D, Bioer Technology, Hangzhou, China) under the following conditions: denaturation at 94°C for 30 s, annealing at 61°C for 30 s, and extension at 72°C for 30 s. The final extension cycle of 72°C was for 5 min. The PCR products were sequenced on an ABI 3100 sequencer (Applied Biosystems, Foster City, CA, USA) and sequence data were analyzed using SeqPilot DNA sequence analysis software (JSI, Kippenheim, Germany).

## PCR-Restriction Fragment Length Polymorphism

Restriction Fragment Length Polymorphism (RFLP) is a technique to discriminate the mutation from wild-type alleles by analysis of patterns derived from cleavage of the DNA. For the RFLP reaction, we used XapI enzyme (Fermentas, USA). Around, 20 U (1 µl) of this enzyme can digest 0.2‐1 µg of PCR product in 15 min. Each 20 µl mixture reaction was prepared with 20 U (1 µl) of XapI enzyme, 100 ng of PCR product, and Green Buffer (10×). The reaction was allowed to incubate at 37 °C for 15 min in a thermocycler then the enzyme was inactivated by heating at 80 °C for 20 min by the manufacturer's instructions. The RFLP products (15 µl) were loaded in 2% agarose gel. GeneRuler DNA Ladder Mix was used as a marker. Post‐staining with EtBr was performed after electrophoresis (100 mV) for a clear identification of different genotypes.

## Tetraplex-Amplification refractory mutation system (Tetraple-ARMS-PCR)

The method employs four different primers to amplify a fragment from DNA containing the variant representing each of the two allelic forms. Primers were designed to amplify fragments of differing sizes for each allele band in order to easily resolve them using agarose gel electrophoresis. The ‘BLAST’ program (<http://www.ncbi.nlm.nih.gov/blast/>) was used to check the specificity of the primers. Details of the primer executed for the present study and the amplicon size for different genotypes were shown in **Supplementary Table 1**. PCR was performed in a total volume of 25 μl containing approximately 50 ng of sample DNA, 2.5 μl of 10× buffer, 2.0 mM MgCl_2_, 0.2 mM dNTPs, 5 pmol of each outer primers (FO and RO), 10 pmol of each inner primers (FN and RM) and 1 U of Taq polymerase (Sigma–Aldrich, USA). In detail, the protocol was 94 °C for 5 min, followed by 35 cycles of 94 °C for 30 s, annealing at 55 °C for 30 s and 72 °C for 30 s, and a final extension at 72 °C for 10 min. The PCR products were separated on 2.0% agarose gel using ethidium bromide.

## Supplementary Table (Analysis statistics WES)

| pedigree ID | Average | % Target bp Covered | | | | | |
| --- | --- | --- | --- | --- | --- | --- | --- |
|  |  | **0X** | **≥ 1X** | **≥ 5X** | **≥ 10X** | **≥ 20X** | **≥ 50X** |
| II.1 | 99.36 | 0.12 | 9.88 | 99.37 | 98.613 | 93.97 | 70.89 |
| II.2 | 80.5969 | 0.139503 | 99.8605 | 99.322 | 97.6565 | 96.560 | 71.5262 |
| II.3 | 131.866 | 0.191 | 99.809 | 99.407 | 98.38 | 94.8026 | 83.271 |
| II.4 | 102.876 | 0.12 | 99.88 | 99.32 | 97.98 | 92.84 | 66.96 |
| III.1 | 117.803 | 0.318 | 99.68 | 99.05 | 97.34 | 90.85 | 65.73 |
| III.2 | 101.82 | 0.14 | 99.86 | 99.32 | 98.11 | 93.53 | 69.93 |
| III.4 | 93.70 | 0.12 | 99.88 | 99.37 | 98.07 | 82.86 | 66.46 |

**References**

1. Spencer DH, Tyagi M, Vallania F, Bredemeyer AJ, Pfeifer JD, Mitra RD, et al. Performance of Common Analysis Methods for Detecting Low-Frequency Single Nucleotide Variants in Targeted Next-Generation Sequence Data. The Journal of Molecular Diagnostics. 2014;16(1):75-88.

2. Stenson PD, Mort M, Ball EV, Howells K, Phillips AD, Thomas NS, et al. The human gene mutation database: 2008 update. Genome Med. 2009;1(1):13.

3. Landrum MJ, Lee JM, Benson M, Brown G, Chao C, Chitipiralla S, et al. ClinVar: public archive of interpretations of clinically relevant variants. Nucleic Acids Res. 2015;44(D1):D862-D8.

4. Karczewski K, Francioli L. The Genome Aggregation Database (gnomAD). MacArthur Lab. 2017.

5. Siva N. 1000 Genomes project. Nature Publishing Group; 2008.

6. Fattahi Z, Beheshtian M, Mohseni M, Poustchi H, Sellars E, Nezhadi H, et al. Iranome: A catalogue of genomic variations in the Iranian population. Hum Mutat. 2019.

7. Adzhubei I, Jordan DM, Sunyaev SR. Predicting functional effect of human missense mutations using PolyPhen‐2. Current protocols in human genetics. 2013;76(1):7.20. 1-7.. 41.

8. Ng PC, Henikoff S. SIFT: Predicting amino acid changes that affect protein function. Nucleic Acids Res. 2003;31(13):3812-4.

9. Schwarz JM, Rödelsperger C, Schuelke M, Seelow D. MutationTaster evaluates disease-causing potential of sequence alterations. Nature methods. 2010;7(8):575.

10. Untergasser A, Cutcutache I, Koressaar T, Ye J, Faircloth BC, Remm M, et al. Primer3—new capabilities and interfaces. Nucleic acids research. 2012;40(15):e115-e.
