# Supplementary material for: Novel manifestations of Warburg micro syndrome type 1 caused by a new splicing variant of RAB3GAP1: a case report
Source: BMC Neurol. 2021 Apr 28;21:180. doi: 10.1186/s12883-021-02204-w (PMC8080372; doi:10.1186/s12883-021-02204-w)
Supplement: Supplementary file 3 — Additional file 3. A list of primers that was used in this study [file 12883_2021_2204_MOESM3_ESM.docx]

**Additional File 3**. a list of primers that was used in this study.

| Gene | Goal | Primers (5ʹ→3ʹ) | Tm (°C) | Amplicon size (bp) |
| --- | --- | --- | --- | --- |
| *XIRP2* | Sanger Sequencing | F*: 5ʹ-GCACCAAGTAGCAGCTCATC-3ʹ | 59.00 | 719 bp |
|  |  | R†: 5ʹ-TTTGAACAGTTCGGTTGGCC-3ʹ | 59.20 |  |
| *MAP3K19* | Sanger Sequencing | F: 5ʹ-ACATTAGCCGAGCATAGTGGC-3ʹ | 60.50 | 526 bp |
|  |  | R: 5ʹ-AGACCTGAGAGTCGGATCCATC-3ʹ | 60.75 |  |
| *RAB3GAP1* | Sanger Sequencing | F: 5ʹ-TCAAAATTACAGCCACTCCTAC-3ʹ | 56.00 | 303 bp |
|  |  | R: 5ʹ-ATGGAGGATATTTATAGGGCAG-3ʹ | 54.50 |  |
| *MAP3K19* | Tetra-primer ARMS‐PCR | FN: 5ʹ-ACATTAGCCGAGCATAGTGGC-3ʹ | 60.50 | 189 bp |
|  |  | RO: 5ʹ-AGACCTGAGAGTCGGATCCATC-3ʹ | 60.75 |  |
|  |  | FO: 5ʹ-ACATTAGCCGAGCATAGTGGC-3ʹ | 60.40 | 382 bp |
|  |  | RM: 5ʹ-TGGAAACTTGTGATTTTGGGGCAC-3ʹ | 62.40 |  |
| *RAB3GAP1* | Tetra-primer ARMS‐PCR | FN: 5ʹ-GCCAGAAGTAAATATACCCTACA-3ʹ | 55.20 | 130 bp |
|  |  | RO: 5ʹ-ATGGAGGATATTTATAGGGCAG-3ʹ | 54.50 |  |
|  |  | FO: 5ʹ-TCAAAATTACAGCCACTCCTAC-3ʹ | 56.10 | 221 bp |
|  |  | RM: 5ʹ-TATTTTGCCATTTTATTGGTACGG -3ʹ | 56.30 |  |
| *RAB3GAP1* | RT-PCR, RT-qPCR | F: 5ʹ-TGAATGACTGGAAACTGATTGGA-3ʹ | 58.10 | 245 bp |
|  |  | R: 5ʹ-TGCTCTTGGAGGAAAGTCATT-3ʹ | 57.20 |  |
| *ALBUMIN (ALB)* | RT-qPCR | R: 5ʹ-TGTTGCATGAGAAAACGCCA-3́ | 59.00 | 244 bp |
|  |  | R: 5ʹ-TGCTCTTTTGTTGCCTTGGG-3́ | 59.25 |  |

***** Forward primer, **†** Reverse primer
