# Supplementary material for: Novel manifestations of Warburg micro syndrome type 1 caused by a new splicing variant of RAB3GAP1: a case report
Source: BMC Neurol. 2021 Apr 28;21:180. doi: 10.1186/s12883-021-02204-w (PMC8080372; doi:10.1186/s12883-021-02204-w)
Supplement: Supplementary file 4 — Additional file 4. Sanger sequencing and conservational analysis for the candidate variants in MAP3K19 and XIRP2 genes. a) Chromatograms shed light on the cosegregation of c.332 T > C of MAP3K19 in the family members. b) Chromatograms showing nucleotide sequences of XIRP2 in the regions of c.9835 T > C. c) MetaDome shows that the c.332 T is located in a region with an average conservational profile in MAP3K19 protein. d) In this XIRP2 tolerance landscape, the region harboring the novel missense variant is partially tolerant in comparison with other parts in this protein. The affected region is located in a somehow variable region [file 12883_2021_2204_MOESM4_ESM.docx]

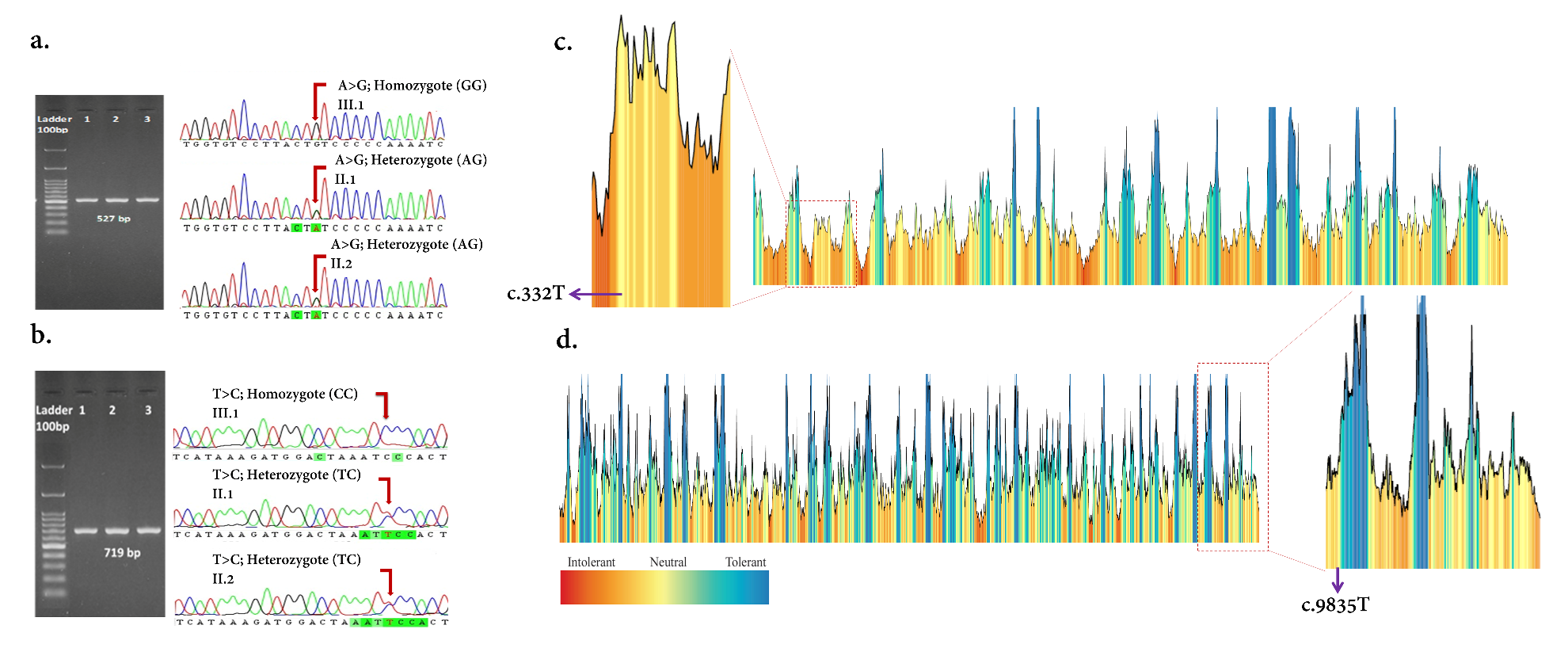


**Additional file 4. Sanger sequencing and conservational analysis for the candidate variants in *MAP3K19* and *XIRP2* genes**. **a)** Chromatograms shed light on the cosegregation of c.332T>C of *MAP3K19* in the family members. **b)** Chromatograms showing nucleotide sequences of *XIRP2* in the regions of c.9835T>C. **c)** MetaDome shows that the c.332T is located in a region with an average conservational profile in MAP3K19 protein. **d)** In this XIRP2 tolerance landscape, the region harboring the novel missense variant is partially tolerant in comparison with other parts in this protein. The affected region is located in a somehow variable region.
