# Supplementary material for: Novel manifestations of Warburg micro syndrome type 1 caused by a new splicing variant of RAB3GAP1: a case report
Source: BMC Neurol. 2021 Apr 28;21:180. doi: 10.1186/s12883-021-02204-w (PMC8080372; doi:10.1186/s12883-021-02204-w)
Supplement: Supplementary file 5 — Additional file 5. 2% agarose gel electrophoresis of tetra primer ARMS-PCR test products for c.332 T > C in the MAP3K19 gene. In this figure: well 1: 100 bp ladder, well 2: negative PCR test control (no template control; NTC), well 3: Homozygous for the normal allele, well 4: Heterozygous for the wild-type allele. The allele frequency was estimated at 0.031 for the c.332 T > C variant [file 12883_2021_2204_MOESM5_ESM.docx]

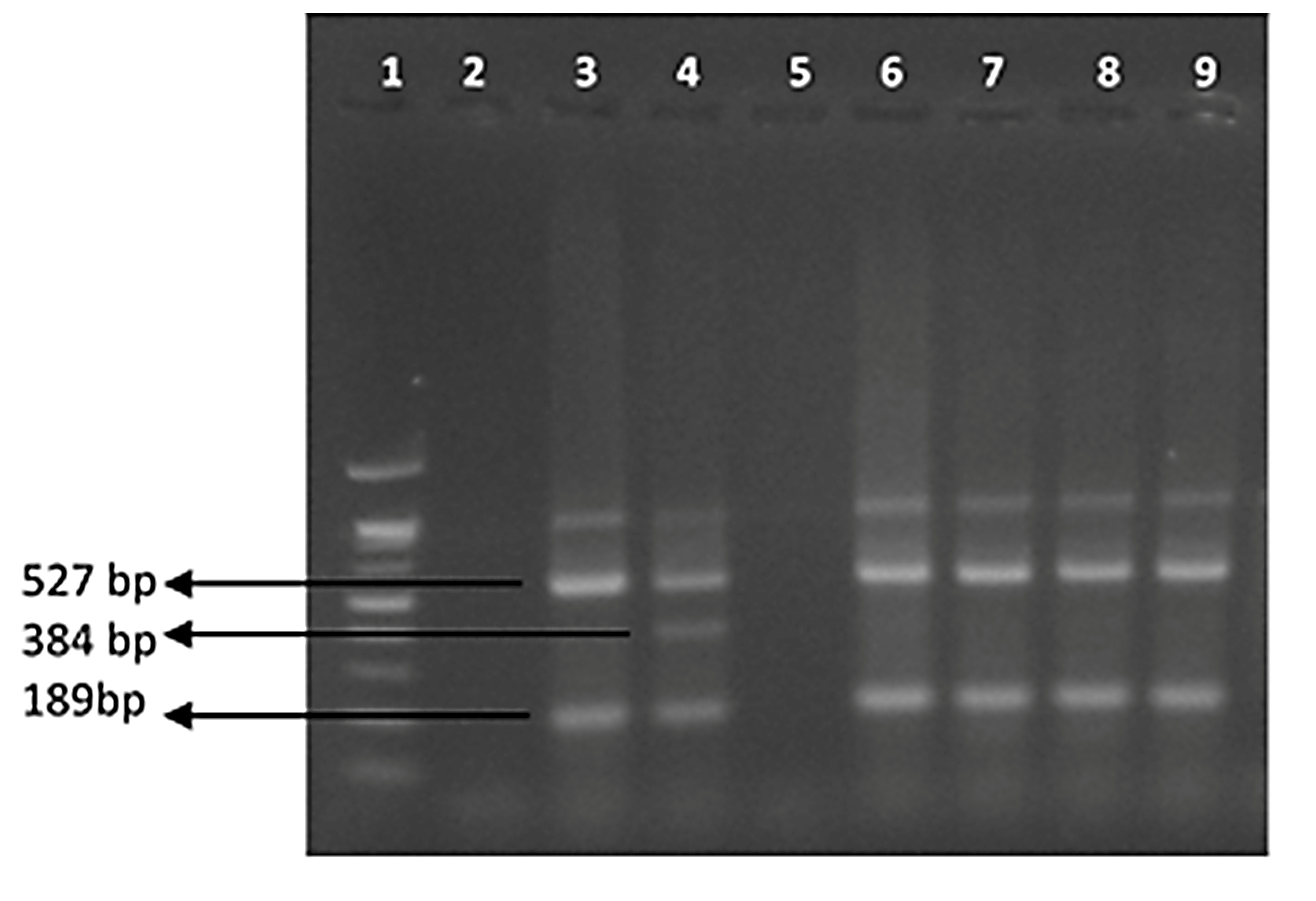


**Additional file 5.** **2%** **agarose gel electrophoresis of tetra primer ARMS‐PCR test products for c.332T>C in the *MAP3K19* gene**. In this figure: well 1: 100 bp ladder, well 2: negative PCR test control (no template control; NTC), well 3: Homozygous for the normal allele, well 4: Heterozygous for the wild-type allele. The allele frequency was estimated at 0.031 for the c.332T>C variant.
