# Supplementary material for: Novel manifestations of Warburg micro syndrome type 1 caused by a new splicing variant of RAB3GAP1: a case report
Source: BMC Neurol. 2021 Apr 28;21:180. doi: 10.1186/s12883-021-02204-w (PMC8080372; doi:10.1186/s12883-021-02204-w)
Supplement: Supplementary file 6 — Additional file 6. A simple and rapid PCR-RFLP assay was used to detect the c.9835 T > C variant in the XIRP2 gene. a) This schematic figure shows the generated fragments after digestion with the XapI restriction enzyme. The variant causes losing the restriction enzyme site. Agarose gel (2.0%) electrophoresis with ethidium bromide staining following the XapI digestion of the PCR products is shown. PCR-RFLP results in normal control showing 340, 249, and 130 bp (T/T: wild-type allele); after XapI digestion, a heterozygous sample will show four distinct bands including 470, 340, 249, and 130 bp. The homozygote reveals three distinct bands consisting of 470, 249, and 130 bp. b) PCR-RFLP assay was used on the samples in order to show the genotyping. In this figure, 1: heterozygote, 2: patient, 3–14, and 16–22 are wild-type alleles [file 12883_2021_2204_MOESM6_ESM.docx]

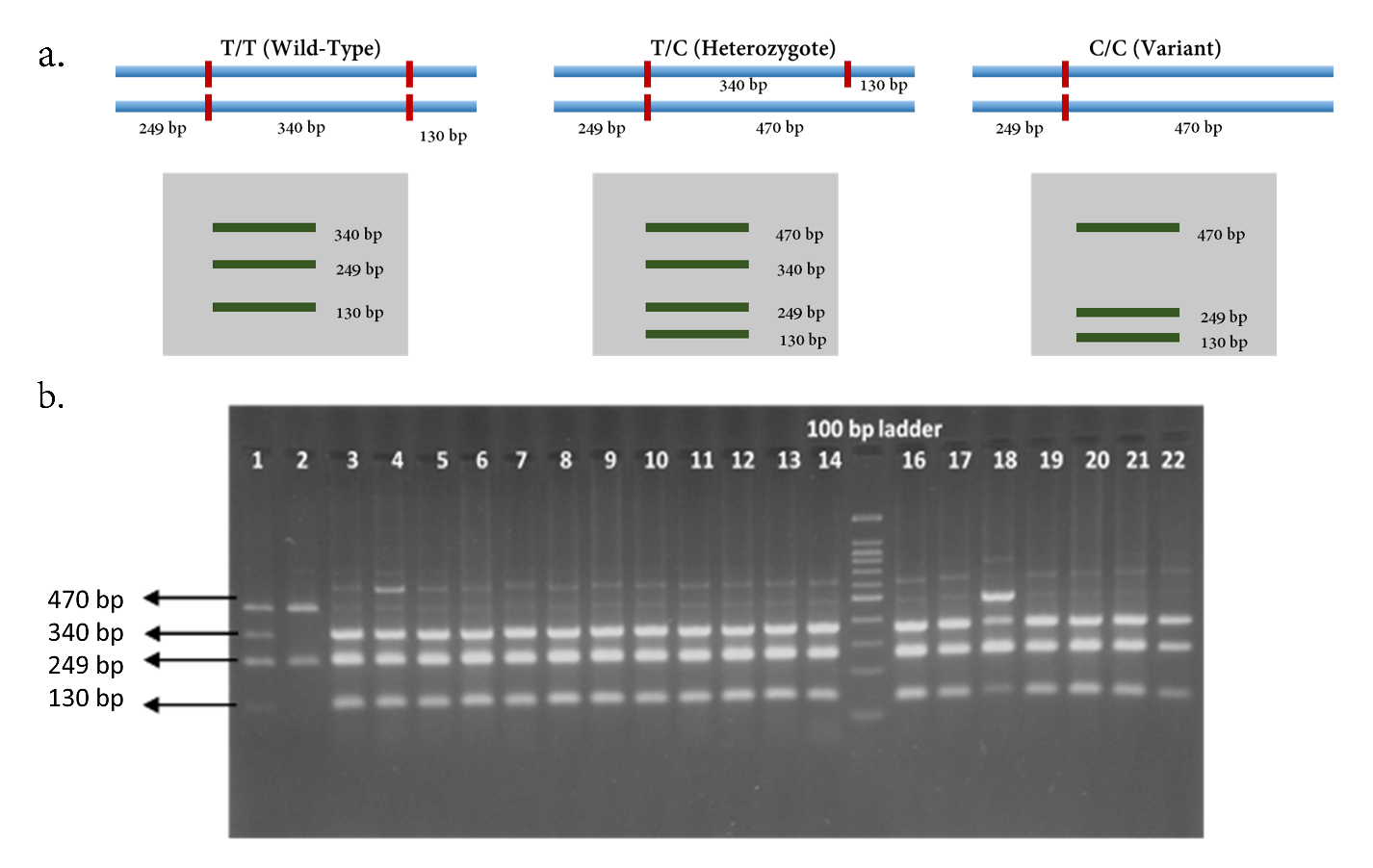


**Additional file 6. A simple and rapid PCR-RFLP assay was used to detect the c.9835T>C variant in the *XIRP2* gene. a)** This schematic figure shows the generated fragments after digestion with the XapI restriction enzyme. The variant causes losing the restriction enzyme site. Agarose gel (2.0%) electrophoresis with ethidium bromide staining following the XapI digestion of the PCR products is shown. PCR-RFLP results in normal control showing 340, 249, and 130 bp (T/T: wild-type allele); after XapI digestion, a heterozygous sample will show four distinct bands including 470, 340, 249, and 130 bp. The homozygote reveals three distinct bands consisting of 470, 249, and 130 bp. **b)** PCR-RFLP assay was used on the samples in order to show the genotyping. In this figure, 1: heterozygote, 2: patient, 3-14, and 16-22 are wild-type alleles.
