# Supplementary material for: Novel manifestations of Warburg micro syndrome type 1 caused by a new splicing variant of RAB3GAP1: a case report
Source: BMC Neurol. 2021 Apr 28;21:180. doi: 10.1186/s12883-021-02204-w (PMC8080372; doi:10.1186/s12883-021-02204-w)

Additional file 7. **a)** Gene expression of the *RAB3GAP1* is changing in different brain regions as age increases. **b)** Gene expression of *RAB3GAP1* in different brain regions and sexes. **c)** Boxplots of RAB3GAP1 mRNA expression levels in ten adult brain regions. The expression levels are based on exon array experiments and are plotted on a log2 scale (y-axis). This dataset was generated with Affymetrix Exon 1.0 ST arrays and brain tissue originating from 134 control individuals, collected by the Medical Research Council Sudden Death. The plot also shows significant variation in *RAB3GAP1* expression across the ten brain regions analyzed, such that expression is higher in the temporal cortex than in any other region. In this figure, TCTX: temporal cortex, FCTX: frontal cortex, OCTX: occipital cortex, SNIG: substantia nigra, THAL: thalamus, PUTM: putamen, HIPP: hippocampus, CRBL: cerebellar cortex, MEDU: medulla (specifically the inferior olivary nucleus), WHMT: intralobular white matter, and N: number of samples analyzed for each brain region. These figures shed light on *RAB3GAP1* expression in the different parts of the brain.

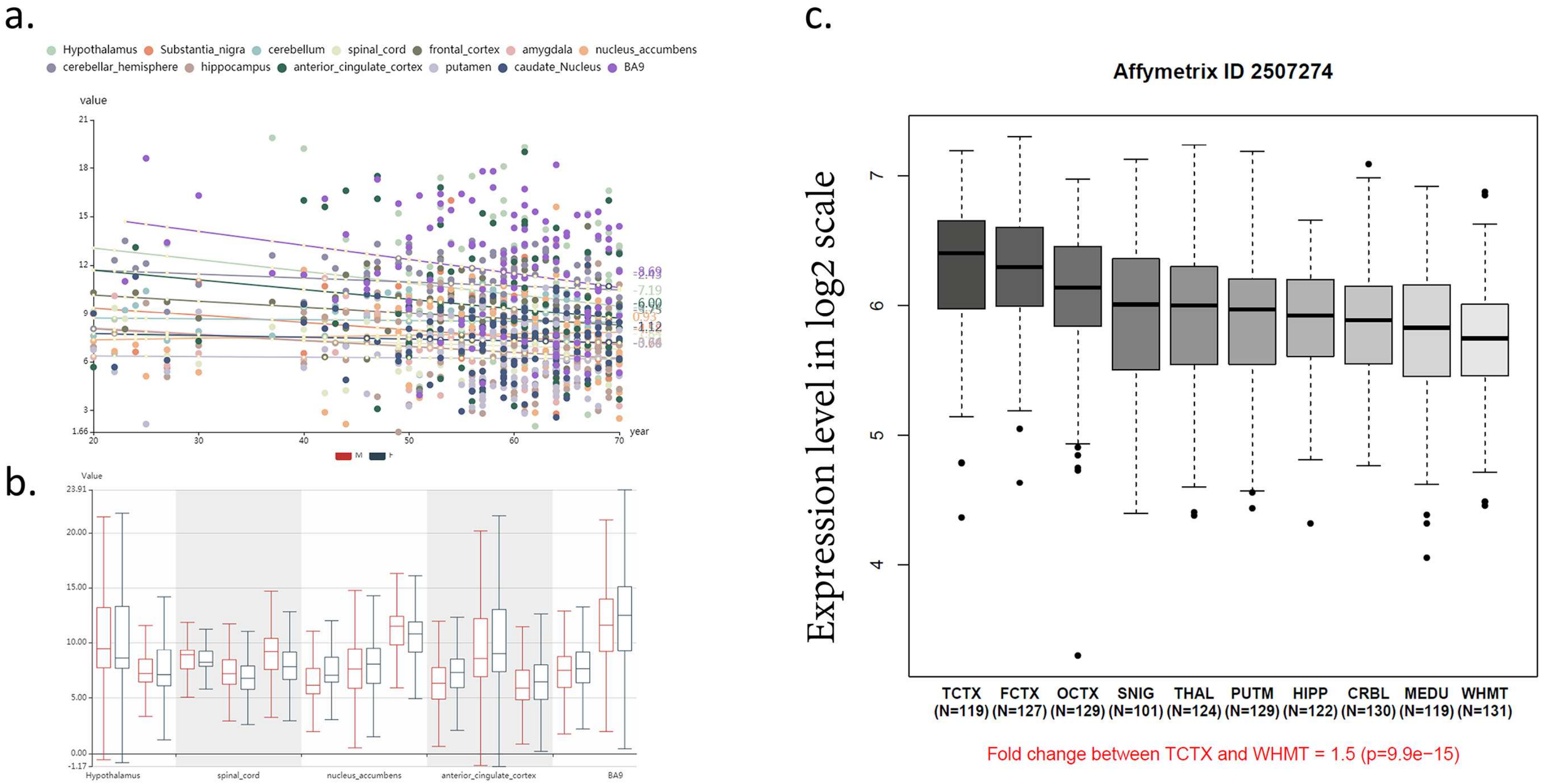

Supplement: Supplementary file 7 — Additional file 7. a) Gene expression of the RAB3GAP1 is changing in different brain regions as age increases. b) Gene expression of RAB3GAP1 in different brain regions and sexes. c) Boxplots of RAB3GAP1 mRNA expression levels in ten adult brain regions. The expression levels are based on exon array experiments and are plotted on a log2 scale (y-axis). This dataset was generated with Affymetrix Exon 1.0 ST arrays and brain tissue originating from 134 control individuals, collected by the Medical Research Council Sudden Death. The plot also shows significant variation in RAB3GAP1 expression across the ten brain regions analyzed, such that expression is higher in the temporal cortex than in any other region. In this figure, TCTX: temporal cortex, FCTX: frontal cortex, OCTX: occipital cortex, SNIG: substantia nigra, THAL: thalamus, PUTM: putamen, HIPP: hippocampus, CRBL: cerebellar cortex, MEDU: medulla (specifically the inferior olivary nucleus), WHMT: intralobular white matter, and N: number of samples analyzed for each brain region. These figures shed light on RAB3GAP1 expression in the different parts of the brain [file 12883_2021_2204_MOESM7_ESM.pdf]
